# Recent Advances in Molecular Magnetic Resonance Imaging of Liver Fibrosis

**DOI:** 10.1155/2015/595467

**Published:** 2015-03-22

**Authors:** Zhiming Li, Jihong Sun, Xiaoming Yang

**Affiliations:** ^1^Department of Radiology, Sir Run Run Shaw Hospital, Zhejiang University School of Medicine, Hangzhou, Zhejiang 310016, China; ^2^Image-Guided Bio-Molecular Intervention Research, Department of Radiology, University of Washington School of Medicine, P.O. Box 358056, Seattle, WA 98109, USA

## Abstract

Liver fibrosis is a life-threatening disease with high morbidity and mortality owing to its diverse causes. Liver biopsy, as the current gold standard for diagnosing and staging liver fibrosis, has a number of limitations, including sample variability, relatively high cost, an invasive nature, and the potential of complications. Most importantly, in clinical practice, patients often reject additional liver biopsies after initiating treatment despite their being necessary for long-term follow-up. To resolve these problems, a number of different noninvasive imaging-based methods have been developed for accurate diagnosis of liver fibrosis. However, these techniques only reflect morphological or perfusion-related alterations in the liver, and thus they are generally only useful for the diagnosis of late-stage liver fibrosis (liver cirrhosis), which is already characterized by “irreversible” anatomic and hemodynamic changes. Thus, it is essential that new approaches are developed for accurately diagnosing early-stage liver fibrosis as at this stage the disease may be “reversed” by active treatment. The development of molecular MR imaging technology has potential in this regard, as it facilitates noninvasive, target-specific imaging of liver fibrosis. We provide an overview of recent advances in molecular MR imaging for the diagnosis and staging of liver fibrosis and we compare novel technologies with conventional MR imaging techniques.

## 1. Introduction

Chronic liver disease is a worldwide health problem, which has a common process-liver fibrosis [1, 2]. There are several etiologies resulting in chronic liver diseases, including chronic infection by hepatotropic viruses (hepatitis B and hepatitis C viruses), chronic exposure to toxins or drugs (e.g., alcohol abuse), chronic alteration of metabolics, and persisting autoimmune reaction. Chronic liver damages may induce both inflammatory response and activation of fibrogenesis. Given persisting fibrogenesis without removal of exposure to the specific etiology, liver fibrosis progresses. Liver fibrosis is characterized by the excess deposition of collagenous extracellular matrix (ECM) components, which often lead to hepatic dysfunction, portal hypertension, and hepatocellular carcinoma [1, 3, 4]. Histologically, liver fibrosis can be classified into a number of different stages, and these stages are directly related to decisions regarding the management of liver fibrosis. Early-stage liver fibrosis can be “reversed” by efficient treatment, while advanced fibrosis and cirrhosis are usually “irreversible” [4–7]. Accurate differentiation of stages is thus critical for efficient management of liver fibrosis. Currently, liver biopsy is the gold standard for diagnosing and staging liver fibrosis. However, due to its invasive nature and relatively high cost, as well as variability in samples and the risk of complications such as procedure-related bleeding and infection, additional biopsies are often rejected by patients after they have initiated treatment, thereby preventing effective long-term follow-up.

Conventional imaging techniques, such as ultrasonography (US), computed tomography (CT), and magnetic resonance imaging (MRI), are noninvasive methods for diagnosing and staging liver fibrosis. However, these techniques are primarily dependent on the detection of morphological alterations in the liver, and these alterations are usually only detected in advanced or late-stage fibrosis, such as cirrhosis [8]. Transient elastography (TE) (fibroscan), which relies on a low-frequency wave generated by a mechanical vibrator, has been used to assess liver fibrosis with relatively high specificity and sensitivity. By using liver stiffness measurement (LSM) value, TE can differentiate patients with no fibrosis from those with mild fibrosis (METAVIR stages F1 and F2) or advanced fibrosis (stages F3 and F4) [9]. Acoustic radiation force impulse (ARFI) imaging is another ultrasound elastography technique, which uses focused high-intensity, short-duration acoustic pulses in order to produce shear waves in the target tissues. According to the findings of a multicenter study, there was a significant correlation between the ARFI measurement and liver fibrosis [10]. MR diffusion-weighted imaging (DWI) can depict the movement of water molecules, but it does not directly reflect the deposition of the ECM. Contrast-enhanced MR imaging (CE-MRI) and MR perfusion-weighted imaging (MR-PWI) rely on the intravenous administration of MR contrast agents that can more precisely reveal hemodynamic changes in the liver. However, these MR techniques are only useful for the diagnosis of advanced liver fibrosis or cirrhosis after long-term hepatic damage. It is therefore essential that a noninvasive, direct, and highly sensitive method for diagnosing early-stage liver fibrosis be developed. Molecular MRI is one such technique, and, in this paper, we present an overview of recent advances in molecular MRI for the diagnosis and staging of liver fibrosis.

## 2. Pathologic Liver Fibrosis and Staging

Liver fibrosis is a common process that occurs in response to liver injury; it is characterized by the excess deposition of ECM. The ECM comprises a group of macromolecules that together form the scaffolding of normal and fibrotic livers. These include collagens, noncollagen glycoproteins, matrix-bound growth factors, glycosaminoglycans, proteoglycans, and matricellular proteins [11]. Liver fibrosis occurs when the rate of ECM synthesis by myofibroblasts exceeds the rate of repair required due to chronic hepatic injuries [4, 12]. Hepatic myofibroblasts mainly stem from resident mesenchymal cells and bone marrow-derived myofibroblasts [1].

**Figure 1:**
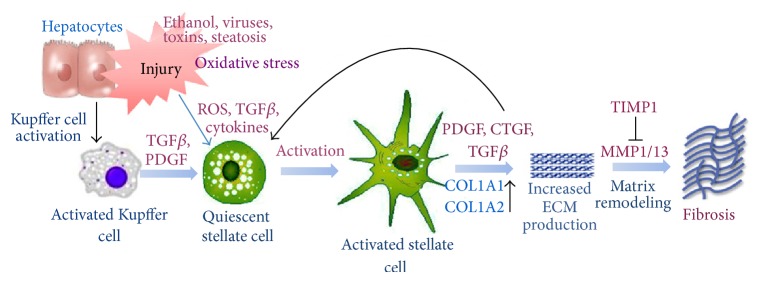
Key agents involved in the activation of hepatic stellate cells during the pathogenesis of liver fibrosis. (Reprinted with permission from [13].)

Hepatic stellate cells (HSCs) are mesenchymal cells that are resident in the liver, and they play a crucial role in fibrogenesis (Figure 1) [13]. Activation of HSCs occurs via a complex process that includes signal transmission, gene expression, and receptor expression. Numerous cytokines are involved in fibrogenesis, including platelet-derived growth factor (PDGF), transforming growth factor-*β* (TGF-*β*), tumor necrosis factor-*α* (TNF-*α*), and interleukin [2, 14].

**Figure 2:**
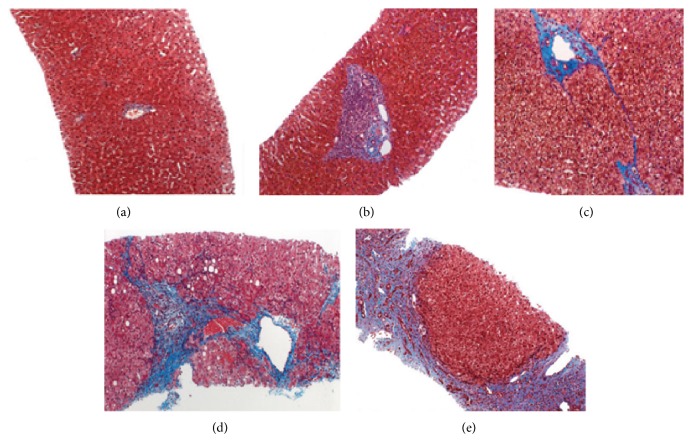
Histological staging of liver fibrosis (photomicrographs of liver biopsy specimens; trichrome stain, ×40): stage F0 (a), stage F1 (b), stage F2 (c), stage F3 (d), and stage F4 (e). (Reprinted with permission from [8].)

Different staging systems are used for the pathological classification of liver fibrosis: the IASL (International Association for the Study of the Liver), Metavir, and Batts-Ludwig systems. These systems have a number of common features, and all basically classify liver fibrosis into five stages (Figure 2): no fibrosis; portal fibrous expansion; thin fibrous septa emanating from portal triads; fibrous septa bridging portal triads and central veins; and cirrhosis [8, 15].

## 3. MRI of Liver Fibrosis

Magnetic resonance imaging is a unique modality that has several advantages over other imaging techniques, including its ability to obtain high resolution images with excellent contrast against a soft tissue background, the flexibility to acquire images using a number of different techniques to facilitate the diagnostic evaluation of organ morphology, physiology, and function, and the ability to project data in an infinite number of imaging planes with no risk of ionizing radiation.

Because it is dependent on the detection of alterations in hepatic morphology, conventional MRI has a high specificity for cirrhosis, but a low sensitivity for earlier stages of liver fibrosis, and it is thus not suitable for staging the disease [8]. Recently, a number of modified MRI-based techniques, including DWI, MRE, PWI, and CE-MRI, have been developed for diagnosing and staging liver fibrosis. These are described in detail below.

### 3.1. MR Diffusion-Weighted Imaging (MR-DWI)

**Figure 3:**
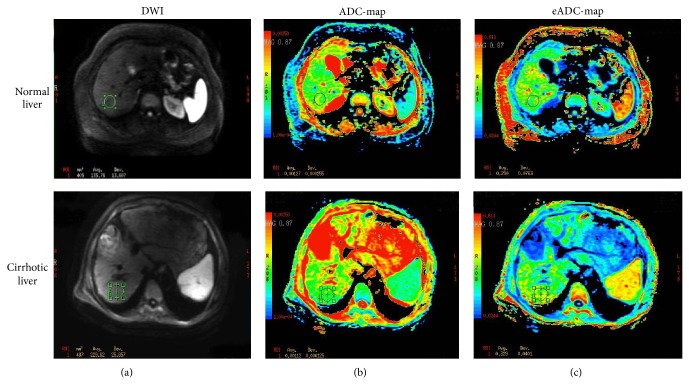
MR-DWI in a normal and cirrhotic liver (*b* value 600 s/mm^2^). DWI images are shown in the far left column. The middle column shows ADC maps superimposed on the corresponding anatomic images. The exponential ADC maps are shown in the far right column. The ADC maps show that the mean ADC value of the cirrhotic liver is lower than that of the normal liver (1.12 × 10^−3^ mm^2^/s versus 1. 27 × 10^−3^ mm^2^/s, resp.).

The MR-DWI technique monitors the Brownian motion of water molecules relative to the temperature and viscosity of the studied environment, and it is routinely used for liver imaging (Figure 3) [16]. Calculation of the apparent diffusion coefficient (ADC) with DWI can facilitate the assessment of liver fibrosis. For example, one recent study showed that ADC values are decreased as the stage of liver fibrosis increases from F0 to F4, but no significant differences in ADC values were detected between stages F0 and F1, as well as F1 and F2 [17]. Another study reported that ADC values were only significantly different between stages F0 and F4 [18]. Together, these findings suggest that DWI is not a reliable method for distinguishing early-stage liver fibrosis [17, 18].

Intravoxel incoherent motion (IVIM) diffusion-weighted (DW) imaging developed for quantitative assessment of the microscopic translational motions of both intracellular and extracellular water molecules, which occur in each voxel of MR imaging [19]. By using IVIM imaging, several factors, such as pure molecular diffusion and microcirculation or blood perfusion, can be distinguished with multiple *b* values [19, 20]. One pilot study demonstrated the usefulness of using IVIM DW imaging with ten *b* factors to determine the difference of pure molecular-based (*D*) and perfusion-related (*D* ^*^, *f*) diffusion parameters, between patients with cirrhosis and patients without liver fibrosis [21]. They found that both ADC and *D* ^*^ were significantly reduced in the cirrhotic liver group compared with those in the healthy liver group, while there was no significant difference between *D* and *f* measurements in the healthy liver and cirrhotic liver groups. Another study showed a significant decrease of both pure molecular diffusion coefficient (*D*_slow_) and perfusion-related diffusion coefficient (*D*_fast_) in the advanced fibrosis group compared to nonadvanced fibrosis group (*P* < 0.05 and *P* < 0.01, resp.) [22]. Furthermore, the IVIM parameters, including pseudo-diffusion coefficient (*D*_*p*_) and perfusion fraction (*f*), can be used for differentiating stages between fibroses ⩾ F2 and F0-1 (*P* < 0.05) [23].

### 3.2. T1*ρ* MR Imaging

T1*ρ* is the spin-lattice relaxation time constant in the rotating frame, which describes the decay of transverse magnetization under the special condition of a spin-lock radiofrequency field [24]. T1*ρ* is sensitive to both low-frequency motional process and static process and thus can be used to investigate macromolecular composition [25]. Because of the fact that liver fibrosis is featured by excess ECM deposition, T1*ρ* MR imaging can be used to assess liver fibrosis. One study using a rat biliary duct ligation model showed that liver fibrosis can be detected with T1*ρ* MR imaging, and the T1*ρ* value increase correlated with liver collagen levels [26]. Another study was conducted with patients of fibrosis stage F4 and healthy volunteers by using T1*ρ* MR imaging, demonstrating that the mean T1*ρ* values increased as Child-Pugh stage increased, and there were significant differences of mean T1*ρ* values among Child-Pugh classes [27]. A recent study further indicated that liver T1*ρ* could be a valuable biomarker for liver injury and fibrosis [28]. Liver T1*ρ* value increased mildly on 48 hours and further increased as the degeneration and necrosis of hepatocytes, while fibrosis appeared and progressed at weeks 2, 4, and 6. In addition, liver T1*ρ* values decreased at weeks 1 and 4 after the withdrawing of the carbon tetrachloride (CCl_4_). These results indicate that T1*ρ* MR imaging is a potential promising technique in monitoring liver injury, as well as liver fibrosis regression and progression.

### 3.3. MR Elastography (MRE)

**Figure 4:**
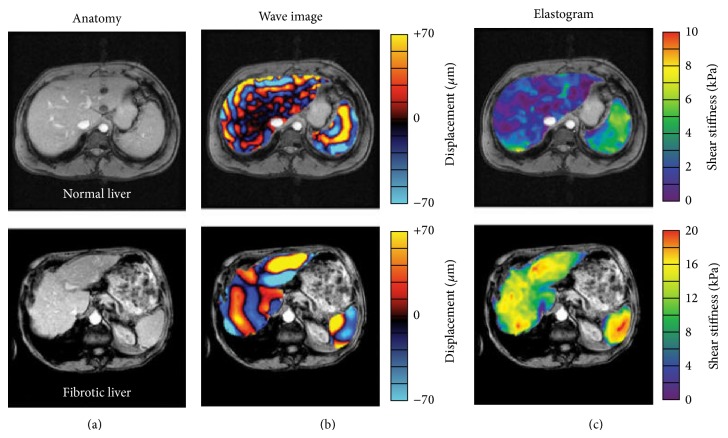
MR elastography images of the livers of a normal volunteer and a patient with cirrhosis. Anatomic images of a normal volunteer and of a patient with grade 4 fibrosis are shown in the far left column (a). The middle column of images shows wave image data in the liver and spleen superimposed on the corresponding anatomic images (b). The resulting elastograms are shown in the far right column (c). The wave images show that the shear wavelength was higher in the fibrotic liver than in the normal liver. The elastograms show that the mean shear stiffness of the fibrotic liver was much higher than that of the normal liver (12.1 ± 1.2 kPa versus 1.8 ± 0.3 kPa, resp.). (Reprinted with permission from [29].)

Magnetic resonance elastography is a state-of-the-art MRI-based technique that can noninvasively quantify the stiffness of the liver by analyzing the propagation of mechanical waves through liver tissue (Figure 4). It is based on the concept that the stiffness of the hepatic parenchyma is increased as fibrosis advances [8, 29, 30]. One study has shown that MRE has a high sensitivity and specificity for detecting liver fibrosis: predicted sensitivity and specificity scores were 91% and 97% for liver fibrosis ≥ stage F2, 92% and 95% for liver fibrosis ≥ stage F3, and 95% and 87% for liver fibrosis ≥ stage F4 [30]. A meta-analysis that compared the effectiveness of MRE and DWI for staging liver fibrosis concluded that MRE was more reliable and resulted in a better combination of sensitivity and specificity, likelihood ratio, diagnostic odds ratio, and area under the summary receiver operating characteristic curve values [31].

However, MRE also has some disadvantages that limit its clinical acceptance: (i) it cannot be performed in livers with high iron overload because of signal-to-noise limitations; (ii) the examination time is longer than that required for ultrasound elastography [32].

### 3.4. MR Perfusion-Weighted Imaging (MR-PWI)

Perfusion-weighted imaging requires intravenous administration of MR contrast agents, and it is used to quantify the microcirculatory status of the liver parenchyma or liver lesions. Perfusion MR parameters are derived from dynamic contrast-enhanced MRI (DCE-MRI) using model-based or model-free techniques [33]. The deposition of collagen in the space of Disse and sinusoidal capillarization result in an increase in the resistance to incoming sinusoidal blood flow [34], which leads to a decrease in portal venous flow to the liver, an increase in hepatic arterial flow, and the subsequent formation of intrahepatic shunts [8]. Transfer of the low-molecular-weight gadolinium contrast medium from the vascular sinusoids into the interstitial space is thus increasingly impeded by liver fibrosis [33]. One dual-input, single-compartment, model-based study reported an increase in absolute arterial blood flow (*F*_a_), arterial fraction (ART), distribution volume (DV), and mean transit time (MTT) and a decrease in portal venous fraction (PV), in patients with advanced liver fibrosis compared with patients with early-stage liver fibrosis [35]. When the DV was used to predict advanced liver fibrosis, the technique had a sensitivity of 76.9% and a specificity of 78.5%. Other researchers have found that when dynamic contrast-enhanced MRI is combined with gadolinium ethoxybenzyl diethylenetriamine pentaacetic acid (Gd-EOB-DTPA), the slope (10–90% ascending slope of the curve) and area under the curve (AUC) are the two best perfusion parameters to use for predicting the severity of liver fibrosis (>F2 versus ⩽F2); *F*_a_ was the best predictor of early liver fibrosis [36].

The MR-PWI technique also has some disadvantages: (i) many factors can affect correlations between perfusion parameters and fibrosis, including cardiac status, fasting state, hepatic congestion, hepatic inflammation, hepatic lesions, and portal venous flow; (ii) an assumption model is required as well as very rapid imaging to reduce image artifacts; (iii) the technique is not suitable for the assessment of structural abnormalities; and (iv) there are a number of technical problems with the technique, including difficulties with image analysis and misregistration corrections [8].

### 3.5. Magnetization Transfer MR Imaging

Magnetization transfer (MT) MR imaging can provide information of reduced signal from macromolecule-rich tissues with exchange of the applied radiofrequency energy between pools of bound and of free protons. MT imaging can be used as a noninvasive technique for imaging collagen and other macromolecules, for example, liver fibrosis. One study with a Niemann Pick type C mouse model showed that collagen deposition was consistent with the observed elevation in MT ratio. There was a 10% increase in collagen content, which produced an increase of MT ratio at approximately 9% [37]. However, due to the complex pathological change of the cirrhotic liver tissues, the MT effects (signal intensity of magnetization transfer contrast (MTC)/signal intensity of non-MTC) were widely variable [38]. Another study reported that the MT ratio was nearly identical between healthy (range 26.0%–80.0%) and cirrhotic livers (range 26.7%–81.2%) by using eight different frequency offsets of the MT pulses [39]. To the best of our knowledge, no literature indicates that MT MR imaging is a valuable technique in assessing liver fibrosis so far.

Thus, conventional MRI techniques, such as DWI, PWI, MRE, and MT imaging, are of limited use for diagnosing and staging liver fibrosis because they are generally focused on morphological or perfusion-related changes in the liver caused by liver fibrosis, rather than molecular changes of fibrosis itself [8], which are the “roots” of fibrotic livers.

## 4. Molecular MR Imaging

Molecular imaging is motivated to directly visualize, characterize, and measure biological processes at the molecular and cellular levels in humans and other living systems, and the techniques include radiotracer imaging/nuclear medicine, MR imaging, MR spectroscopy, optical imaging, ultrasonography, and others [45]. Molecular MR imaging has become a novel technique for assessing specific cellular or subcellular events and is becoming one of the core integrative technologies in biomedicine [46]. In contrast to US, CT, and PET (positive emission tomography) or SPECT (single photon emission computed tomography), molecular MR imaging has several superior advantages, including offering high spatial resolution images, simultaneously extracting anatomic, physiologic, and functional information [47], and more importantly avoiding harmful ionizing radiation.

The strength of MR signals depends upon the longitudinal (T1) and transverse (T2) proton relaxation times of water, and thus differences in proton relaxation times cause various contrasts on MR images. To maximize image quality, MR contrast agents are often needed to decrease T1 and T2 relaxation times. In general, there are two types of MR contrast agents: paramagnetic and superparamagnetic compounds. Paramagnetic contrast agents, also called T1 or positive contrast agents, are usually composed of Gadolinium^3+^ or Mn^2+^, which generates positive signals on T1-weighted images. Superparamagnetic contrast agents, also called T2 or negative contrast agents, are usually constructed with iron oxide, which generates negative signal or signal void on T2- and T2^*^-weighted images [48].

The specific contrast agents for molecular MR imaging are defined as “probes used to visualize, characterize, and measure biological processes in living systems. Both endogenous molecules and exogenous probes can be molecular imaging agents” [45]. These MR imaging probes are usually constructed by different nanoparticles that contain paramagnetic or superparamagnetic metals, such as nanoscaffolds loaded with gadolinium chelates or nanoparticles carrying superparamagnetic iron oxide.

In general, systemically administrated molecular MRI probes are target-specific, which depend on the ligands conjugated onto the MRI probes. These ligands can specifically target molecules overexpressing at the diseased site or lesion. The ligands can be monoclonal antibodies or their fragments, peptides, small molecular peptidomimetics, vitamins, or aptamers. The target-specific molecular MRI probes function by following the mechanism of ligand-molecule binding, that is, the specific interaction of ligands with their corresponding molecules of the targets (such as receptors expressed on cell surfaces) to form an antigen-antibody pair like complex [49].

As mentioned above, the molecular MR imaging of liver fibrosis is based on the development of contrast agents, known as activatable MR imaging probes to elicit detectable MR signal changes in response to the local environment or to “sense” specific molecular states [50]. The contrast agents are usually designed and synthesized as a category of nanoparticulate probes composed of molecular targets and contrast-generating metals. The probes of detecting liver fibrosis ought to have the capability of specifically targeting and binding ECM, of which excessive accumulation can result in fibrosis. ECM is under the dynamic balance of synthesis and degradation in the normal liver. ECM is a normal component of Glisson's capsule, portal tracts, central veins, and the subendothelial space of Disse and accounts for less than 3% of the relative area on a liver tissue section and approximately 0.5% of the wet weight [14]. In the fibrotic liver, remarkable changes present in ECM quantitatively and qualitatively.

A number of molecules are present in increased amounts in fibrotic livers, including fibrillar collagens (types I, III, and IV), some nonfibrillar collagens (types IV and VI), a number of glycoproteins (cellular fibronectin, laminin, SPARC, osteonectin, tenascin, and von Willebrand factor), proteoglycans, and glycosaminoglycans (perlecan, decorin, aggrecan, lumican, and fibromodulin). Of these molecules, the fibrillar collagens (especially types I and III) and elastin are the most abundant in the ECM [51]. Accordingly, the type of ECM in the space of Disse changes from a normal, low-density, basement membrane-like matrix primarily composed of types IV and VI collagens to a matrix primarily composed of interstitial type I and III collagens and fibronectin [14]. Thus, the components of the ECM are potential important cellular and molecular targets for molecular MRI to diagnose and stage liver fibrosis.  Table 1 summarizes ligand-molecule pairs that have previously been used for molecular MRI to diagnose and stage liver fibrosis.

## 5. Application of Molecular MRI in Liver Fibrosis

Molecular MRI of liver fibrosis has been used to directly detect molecular changes in the ECM and HSCs.

### 5.1. EP-3533 Probe Targeting Type I Collagen

**Figure 5:**
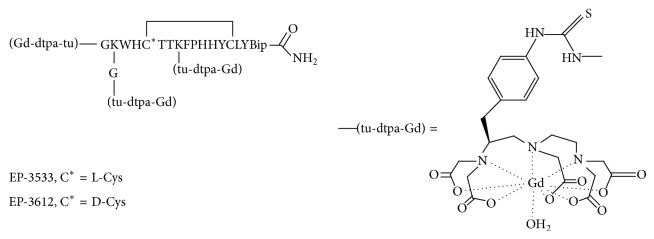
Collagen-targeting contrast agent: L-amino acids are designated by letter, except where otherwise noted; Gd chelates are appended through the N terminus, through branched Lys-Gly residues at the N terminus, and through a Lys side chain within the cyclic portion of the peptide. (Reprinted with permission from [52].)

Compared with the normal liver, the amount of type I collagen in fibrotic livers is significantly increased (from 36% to 53%) [53]. Therefore, type I collagen can be used as a molecular target for detecting liver fibrosis by molecular MRI. EP-3533 (gadolinium-diethylenetriamine pentaacetic acid-GKWHCTTKFPHHYCLY) is a type I collagen-targeting MR contrast agent that has previously been used for myocardial infarction [54, 55], pulmonary fibrosis [56], and liver fibrosis [40, 41]. It is composed of a 16-amino-acid peptide that has three amino acids flanking each side of a cyclic peptide of 10 amino acids joined by disulfide bonds (Figure 5) [52]. The peptide contains three primary amines (the N terminus and two lysine side chains), and these are used to append three gadopentetate dimeglumine moieties via a thiourea linkage [54]. The relaxivity of EP-3533 is 16.1 mM^−1^ s^−1^ per Gd^III^ ion at 1.41 Tesla and 37°C (PBS) or 5.4 mM^−1^ s^−1^ per Gd^III^ ion at 4.7 Tesla and 25°C (PBS) [57].

Molecular MRI has been trialed in two animal models of liver fibrosis [40]: (i) a diethylnitrosamine (DEN) rat model, which is created by feeding rats 100 mg/kg/week DEN for 4 weeks, which results in moderate to advanced liver fibrosis (Ishak scores 3–6); and (ii) the carbon tetrachloride (CCl_4_) mouse model, which is created by feeding mice 0.1 mL of a 40% solution of CCl_4_ in olive oil three times a week for 20 weeks, which also results in moderate to advanced liver fibrosis (Ishak scores 3–6). EP-3533 is administered intravenously and a nontargeted gadolinium-diethylenetriamine pentaacetic acid (Gd-DTPA) is used as a control. The cyclic peptide of EP-3533 has a specific affinity for type I collagen, whereas the gadolinium moieties generate strong T1 MR signals. By correlating MR imaging, tissue analysis, and real-time polymerase chain reaction (PCR) findings, it was concluded that molecular MRI of liver fibrosis with the EP-3533 collagen-targeting probe was capable of identifying fibrotic tissues in both the DEN rat and CCl_4_ mouse models.

Another study attempted to use EP-3533-based molecular MRI to detect type I collagen in liver fibrosis in the CCl_4_ mouse model [41]. By comparing the technique with other conventional MRI methods, the authors found that there was a strong positive linear correlation between the muscle contrast-to-noise ratio (ΔCNR) and liver hydroxyproline levels (hydroxyproline is an amino acid [C_5_H_9_NO_3_] that is a natural constituent of collagen) as well as the ΔCNR and conventional Ishak fibrosis scoring, which indicates the potential usefulness of this technique for staging liver fibrosis.

### 5.2. CLT1 Peptide Probe Targeting Fibrin-Fibronectin Complexes

The accumulation of fibrin in the liver occurs during acute as well as chronic experimental liver injury [59]. Fibronectin is a type of structural glycoprotein present in the liver ECM [53]. Fibrin-fibronectin complexes exist in fibrotic livers because of cross-linkages between fibrin/fibrinogen and fibronectin [59]. Some authors have shown that the CLT1 and CLT2 peptides can specifically bind to fibrin-fibronectin complexes in the ECM of different tumors with little binding to normal tissues, suggesting that CLT peptides may bind to an epitope in the fibrin-fibronectin complex formed as a result of plasma clotting within tumors and at sites of tissue injury [60]. It is thus feasible that liver fibrosis can be detected by using the CLT1 peptide as a probe for targeting fibrin-fibronectin complexes. One group of researchers synthesized CLT1 peptide-targeting nanoglobular contrast agents by conjugating gadoteric acid (Gd-DOTA) and peptides on the surfaces of generation 2 (G2) and generation 3 (G3) nanoglobules (lysine dendrimers with a cubic silsesquioxane core) [61]. Approximately two peptides and 25 Gd-DOTA chelates have reportedly been conjugated onto the surfaces of 32 amine groups of the G2 nanoglobule, and 3 peptides and 43 Gd-DOTA chelates have been conjugated onto the surfaces of 64 amine groups of the G3 nanoglobule. The T1 relaxivities of peptide-targeted G2 and G3 nanoglobules are 7.92 mM^−1^ s^−1^ and 8.20 mM^−1^ s^−1^, respectively, at 3 Tesla. Other studies have shown that a CLT1 peptide-targeting contrast agent, CLT1-(Gd-DTPA), which is conjugated to a cyclic decapeptide, CGLIIQKNEC (CLT1), and Gd-DTPA can be used for molecular MRI of fibrin-fibronectin complexes in tumor tissues, with CLT1-(Gd-DTPA) T1 and T2 relaxivities of 4.22 mM^−1^ s^−1^and 4.45 mM^−1^ s^−1^, respectively, at 3 Tesla [62].

Knowledge of CLT1-binding to fibrin-fibronectin complexes has been adapted for the evaluation of liver fibrosis, and some authors have produced a cyclic decapeptide CLT1-targeting contrast agent for molecular MRI of liver fibrosis [42], based on CLT1's specific binging to the fibrin-fibronectin complex [60]. A CLT1 peptide-targeting nanoglobular contrast agent (Gd-P) was used for dynamic molecular MRI of a mouse model to detect and characterize liver fibrosis at 7.0 Tesla. Compared with the control KAREC-conjugated nanoglobular contrast agent (Gd-CP) and a nontargeting nanoglobular contrast agent (Gd-C), different enhancements were observed between normal and fibrotic livers when Gd-P was used, which is indicative of the usefulness of this molecular MRI approach employing a CLT1 peptide-targeting nanoglobular contrast agent for the detection and staging of liver fibrosis [42].

### 5.3. ESMA Probe Targeting Elastin

Elastin is a type of noncollagenous protein found in the ECM that is secreted by HSCs; it is also associated with the stage of liver fibrosis. Elastin is an essential component of the ECM and elastic fibers, and, together with fibrillins, it provides resilience and elastic recoil in tissues. ESMA (BMS-753951) is an elastin-specific MR contrast agent that has previously been used for assessing atherosclerotic plaques [58] and coronary wall remodeling [63]. It is a Gd-DTPA chelate that is linked to the D-amino acid D-phenylalanine to form a low-molecular-weight MR contrast agent with moderate specificity for elastin. ESMA has a similar blood half-life to current commercially available extracellular Gd-based MR contrast agents [63].

**Figure 6:**
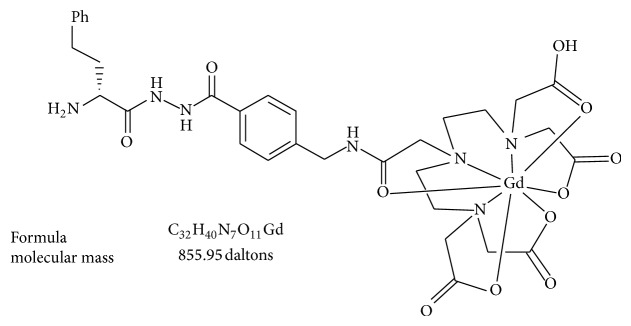
Structural diagram of ESMA showing the molecular mass. (Reprinted with permission from [58].)

The feasibility of monitoring ECMs with ESMA (Figure 6), the elastin-specific MR contrast agent, has previously been assessed, and the study findings suggested that elastin-based molecular MRI has potential as a noninvasive method for monitoring ECM remodeling during liver fibrosis [43].

### 5.4. RGD Peptide Probe Targeting Integrins

As noted above, HSCs play an important role in liver fibrogenesis. Integrins are a special type of heterodimeric transmembrane receptor expressed in HSCs. They are composed of *α* and *β* subunits that transduce signals from the ECM to HSCs and other mesenchymal cell types [64]. Integrins are upregulated in fibrotic liver disease or during HSC activation [65, 66], and *α*_v_ *β*_3_-integrin is highly expressed in HSCs [44, 67, 68]. As cell surface receptors, integrins mediate communication between cells and the ECM, and they appear to play a major role in the development of profibrogenic effects since integrin-linked adhesions (cell-cell and cell-matrix) can promote the migration and proliferation of HSCs and inhibit their apoptosis during liver fibrogenesis [68, 69]. A common feature of integrins such as *α*_v_ *β*_3_ is that they bind to ECM proteins via a three-amino-acid sequence, RGD (arginine-glycine-aspartic acid).

A previous review has provided a detailed examination of molecular MRI targeting integrin *α*_v_ *β*_3_ and the RGD peptide [70]. Most of the literature on RGD-mediated molecular MRI has focused on tumors, and there are few reports on its efficacy in liver fibrosis. Two RGD-based molecular imaging probes have been reported previously. One consists of cRGD conjugated to Gd-DOTA; it has a relaxivity of 9 mM^−1^ s^−1^ at 1.41 Tesla and 40°C and can bind the *α*_IIb_ *β*_3_-receptor as well as the *α*_v_ *β*_3_-receptor. The other consists of cRGD conjugated to Gd-DOTA and is used for selective imaging of *α*_v_ *β*_3_-integrin; it has a relaxivity of 7.4 mM^−1^ s^−1^ at 1.5 Tesla and 25°C [57].

Other researchers have used the cyclo peptide c(RGDyC) to bind *α*_v_ *β*_3_-integrin-conjugated ultrasmall superparamagnetic iron oxide to form USPIO, a T2 contrast agent. This probe (c(RGDyC)-USPIO) has then been used to specifically target activated HSCs [44]. Rats with normal and fibrotic livers were subjected to MR scanning with either c(RGDyC)-USPIO or USPIO, and it was found that the reduction in T2 relaxation times in fibrotic rats was much greater with c(RGDyC)-USPIO than USPIO.

### 5.5. Biodistribution and Clearance of Targeted Molecular Imaging Probes

The biodistribution and clearance of molecular imaging probes depend on their sizes. By using a mouse model, some authors investigated the biodistribution of EP3533 at 15 min after its systemic administration, showing the biodistribution as nmol Gd per gram wet tissue: kidney (223), spleen (77.3), liver (50.4), and lung (29.1) [52]. The blood half-life of EP3533 was 19 ± 2 min, and EP3533 was largely eliminated from the mouse body by 24 hours [41]. One study showed that CLT1-targeted G2 nanoglobular led to its much lower tissue accumulation compared to the targeted G3 agent 48 hours after the systemic administration, because of smaller sizes and less CLT1 peptides conjugated with the G2 agent [61]. Due to a smaller size, the targeted G2 nanoglobular contrast agent cleared more rapidly from the body than the relatively larger G3 agent. In addition, some authors reported the fact that a molecular imaging probe, c(RGDyC)-USPIOs, distributed more extensively in the perisinusoidal space of Disse where the HSCs resided preferentially in the fibrotic liver [44].

One recent study demonstrated a rapid biliary excretion of relative large nanoparticles (~250 nm), while nanoparticles less than 5~6 nm can be bioeliminated through the kidneys. Such small-size nanoparticles can be also cleared by the mononuclear phagocyte system and may be metabolized further or eliminated via bile, urine, or respiration [71].

## 6. Conclusion

Liver fibrosis is a common process that occurs in response to chronic liver injuries. While conventional MRI techniques are useful for assessing advanced liver fibrosis, molecular MRI may be a more valuable tool for noninvasive detection of early-stage liver fibrosis. Recent studies of molecular MRI of liver fibrosis have been confined to producing target-specific molecular MRI probes (such as iron oxides) that can specifically target certain components of the ECM or HSCs in early-stage fibrotic livers. Moreover, nuclear imaging techniques have been used for achieving of molecular information. Radioisotope can be conjugated to a target-specific probe, which thereby can specifically bind targeting molecules in vivo. As the development of molecular imaging agents, nuclear imaging using ECM-specific probes may become a valuable technique for assessing liver fibrosis. Although molecular MRI of liver fibrosis is still at its developing phase, the conception of a target-specific molecular MRI approach is opening new avenues for effective management of this life-threatening disease.

## Figures and Tables

**Table 1:** Studies that have assessed molecular MRI of liver fibrosis.

Study/year	Targeted molecules	Targeting probes	Animal model	MR effect	Significance
Polasek et al., 2012 [40]	Type I collagen	EP-3533	The rat DEN model & the CCl_4_ mouse model	Shortening the T1 relaxation time	Identifies fibrotic tissue in animal models of liver fibrosis
Fuchs et al., 2013 [41]	Type I collagen	EP-3533	The CCl_4_ mouse model	Shortening the T1 relaxation time	Diagnoses and stage sliver fibrosis in an animal model
Chow et al., 2013 [42]	Fibrin-fibronectin	CLT1-peptide-targeted nanoglobular contrast agent (CLT1(Gd-DOTA))	The CCl_4_ mouse model	Shortening the T1 relaxation time	Detected and stage sliver fibrosis by probing the accumulation of fibronectin
Ehling et al., 2013 [43]	Elastin	ESMA	The CCl_4_ mouse model	Shortening the T1 relaxation time	Elastin-based molecular MRI may be suitable for noninvasive monitoring of ECM remodeling during liver fibrosis
Wang et al., 2011 [44]	*α*_v_ *β*_3_-Integrin	c(RGDyC)-USPIO	The CCl_4_ mouse model	Shortening the T2 relaxation time	Targets HSC imaging with c(RGDyC)-USPIO

## Abbreviations

US: Ultrasonography
CT: Computed tomography
CTPI: Computed tomography perfusion imaging
MR-DWI: Magnetic resonance diffusion-weighted imaging
IVIM: Intravoxel incoherent motion
MRE: Magnetic resonance elastography
MR-PWI: Magnetic resonance perfusion-weighted imaging
ECM: Extracellular matrix
MRI: Magnetic resonance imaging
CE-MRI: Contrast-enhanced magnetic resonance imaging
LSM: Liver stiffness measurement
ARFI: Acoustic radiation force impulse
HSC: Hepatic stellate cell
PDGF: Platelet-derived growth factor
TGF-*β*: Transforming growth factor-*β*
TNF-*α*: Tumor necrosis factor-*α*
IASL: International Association for the Study of the Liver
ADC: Apparent diffusion coefficient
DCE-MRI: Dynamic contrast-enhanced magnetic resonance imaging
*F*_a_: Absolute arterial blood flow
ART: Arterial fraction
DV: Distribution volume
MTT: Mean transit time
PV: Portal venous fraction
Gd-EOB-DTPA: Gadolinium ethoxybenzyl diethylenetriamine pentaacetic acid
MT: Magnetization transfer
AUC: Area under the curve
PET: Positive emission tomography
SPECT: Single photon emission computed tomography
DEN: Diethylnitrosamine
CCl_4_: Carbon tetrachloride
Gd-DTPA: Gadolinium-diethylenetriamine pentaacetic acid
PCR: Polymerase chain reaction
Gd-DOTA: Gadoteric acid.
